# Non-suicidal Self-Injury and Suicide Attempts: A Secondary Analysis Describing the Patterns and Clinical Characteristics of Patients Presenting With Self-Harm to a Tertiary Care Hospital

**DOI:** 10.7759/cureus.80715

**Published:** 2025-03-17

**Authors:** Kashyap Shah, Rahul Mathur, Saloni Mishra, Shivani Dua, Varchasvi Mudgal

**Affiliations:** 1 Psychiatry, Mahatma Gandhi Memorial Medical College, Indore, IND; 2 Psychiatry, Kalinga Institute of Medical Sciences, Bhubaneswar, IND

**Keywords:** non-suicidal self-injury, nssi, self-harm, suicidal attempt, suicide

## Abstract

Background

Self-harm includes suicide attempts and non-suicidal self-injury (NSSI), both of which are linked to psychiatric disorders and psychosocial stressors. While suicide attempts involve an intent to die, NSSI often serves as a maladaptive coping mechanism. In India, stigma and limited mental health resources hinder early intervention. This study analyzes self-harm patterns, psychiatric comorbidities, and risk factors in patients presenting to a tertiary care hospital.

Methodology

This retrospective study reviewed the medical records of 165 patients with suicide attempts or NSSI between January and June 2024. Data on demographics, psychiatric diagnoses, self-harm methods, and substance use were analyzed using descriptive and inferential statistics.

Results

Of the 165 cases, 69 involved suicide attempts, and 96 involved NSSI. Suicide attempts were more common among individuals aged 31-40, whereas NSSI was predominant in the 18-30 age group. Depressive disorder was the most frequent diagnosis among those attempting suicide, affecting 55 (68.7%) individuals, while substance use disorder was more prevalent in NSSI cases, with 51 (67.1%) individuals affected. Self-poisoning emerged as the most common method, possibly influenced by weak pesticide regulations in India. A significant association was found between suicide intent and a history of past suicide attempts.

Conclusion

Early screening, access control to harmful substances, identifying at-risk populations, and structured post-discharge care are essential in reducing self-harm and suicide risk. Targeted interventions can improve mental health outcomes in at-risk populations.

## Introduction

Suicide remains a significant public health issue, with over 700,000 deaths annually and an estimated 20 suicide attempts per death [1]. While well-established risk factors include psychiatric disorders like depression and alcohol use disorder, many suicides occur impulsively during crises, highlighting the complexity of self-harm behaviors. Self-harm encompasses a spectrum of behaviors, broadly categorized into self-harm by suicide and non-suicidal self-injury (NSSI), depending on the presence or absence of suicide intent. Both are linked to psychiatric conditions and socio-environmental stressors, making them a global concern [2].

NSSI involves deliberate self-inflicted harm, such as cutting or burning, without the intent to die. It is particularly prevalent among adolescents and young adults, with estimated rates ranging from 18 to 22% [3]. Often associated with psychiatric conditions, especially mood and anxiety disorders, NSSI is sometimes used as a coping mechanism to manage distressing emotions. While it may provide temporary relief, it also increases the risk of suicidal ideation and behavior over time [4]. Previous research suggests that repeated NSSI can lead to desensitization to pain, potentially escalating to more lethal self-harm or suicide attempts [2]. Despite lacking direct suicide intent, NSSI is linked to future suicide risk, psychiatric comorbidities, and significant psychosocial impairment [5].

The distinction between NSSI and suicide attempts remains complex, necessitating further research to identify distinctive features. In India, self-harm is particularly concerning due to social stigma, which often prevents individuals from seeking timely support. Fear of judgment and social exclusion can lead to isolation and family distress, further increasing the likelihood of repeated self-harm. Addressing these issues requires culturally sensitive interventions that reduce stigma, promote awareness, and encourage individuals to seek help without fear.

Given the complexity of self-harm behaviors, objective research is essential to understand the patterns and risk factors associated with NSSI and suicide attempts. This study aims to examine their relationship by analyzing sociodemographic factors, psychiatric symptoms, substance use, and past self-harm attempts. Identifying at-risk populations and clinical characteristics can provide valuable insights into the progression from NSSI to suicide attempts, enabling targeted prevention strategies and improved mental health interventions.

## Materials and methods

The study design is a secondary analysis of patients diagnosed with a suicide attempt and NSSI. The sample was selected based on the type of self-harm, i.e., suicide attempt or NSSI, and any associated psychiatric disorder. The medical records of the selected patients were reviewed, ensuring data quality and confidentiality, and relevant information was extracted. Approval from the ethics and scientific review committee of the tertiary care hospital was obtained (Appendix 1).

The inclusion criteria for this study consisted of patients aged 18 years and above who presented to a tertiary care hospital in Central India. These patients were recorded in the Consultation Liaison register of the psychiatry department presenting with NSSI or suicide attempts. Eligible participants were those whose relevant psychiatric records on either NSSI or suicide attempts were available for the study period between January 2024 and June 2024. Additionally, all diagnoses were made per the ICD-10 criteria. All data have been anonymized to ensure patient confidentiality.

The exclusion criteria for this study included patients whose medical records had missing or incomplete data regarding NSSI or suicide attempts. Patients attempting self-harm during a hospital stay, those who presented with accidental injuries, or other types of injuries that did not amount to NSSI or a suicide attempt were also excluded.

**Figure 1:**
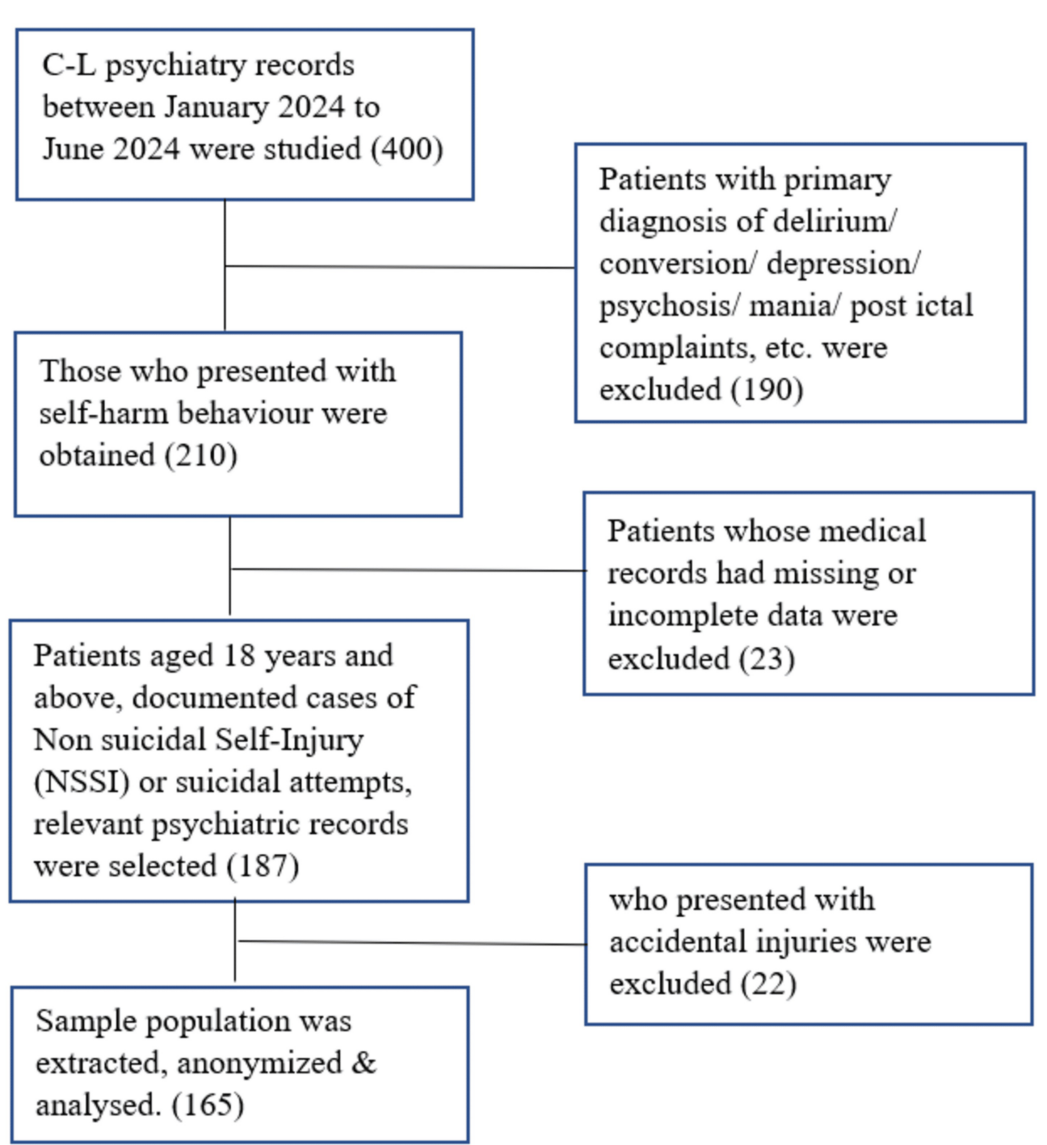
Diagrammatic representation of the methodology.

Data extraction included demographic variables such as age, gender, marital status, etc., and clinical variables such as primary medical diagnosis, comorbid psychiatric diagnoses, history of NSSI and suicide attempts (frequency, motives, outcomes, etc.), psychiatric symptoms (depression, anxiety, etc.), substance use (alcohol, tobacco, etc.), and treatment history (medication, psychotherapy, hospitalization, etc.). The collected data were analyzed using Microsoft Excel 2023, Version: 18.2409. Descriptive and inferential statistics were used for categorical and continuous variables (Figure 1).

## Results

Among the study's population of 187 patients, 165 (88.7%) engaged in intentional self-harm, while a smaller subset of 22 (12.3%) reported accidental self-harm and was therefore excluded (Table 1). Consequently, the final sample population consisted of 165 patients (88.7%).

**Table 1:** Demographic and clinical parameters.

Socio-demographic and clinical parameters	Suicide attempt, N = 69 (%)	NSSI, N = 96 (%)	Total (Out of 165)
Gender			
Male	46 (66.7)	56 (58.3)	102
Female	23 (33.3)	40 (41.7)	63
Age group (years)			
18-30	16 (23.2)	74 (77)	90
31-40	30 (43.5)	12 (12.5)	42
41-50	16 (23.2)	8 (8.3)	24
51-60	4 (5.8)	2 (2.1)	6
>60	3 (4.3)	0 (0)	3
Marital status			
Married	11 (16)	21 (21.9)	32
Unmarried	32 (46.3)	44 (45.8)	76
Separated/Divorced/Widowed	26 (37.7)	31 (32.3)	57
Referring department			
Emergency medicine	28 (40.6)	73 (76)	101
Medicine	29 (42)	16 (16.7)	45
Surgery	9 (13)	4 (4.2)	13
ENT	1 (1.4)	2 (2.1)	3
Orthopaedics	2 (2.9)	0 (0)	2
Ophthalmology	0 (0)	1 (1)	1
Mode of self-harm			
Ingestion of harmful substance	33 (47.8)	71 (74)	104
Hanging	25 (36.2)	0 (0)	25
Using sharp objects	0 (0)	22 (22.9)	22
Burning	10 (14.5)	1 (1)	11
Others	1 (1.4)	2 (2.1)	3

The study analyzed 69 (41.8%) cases of suicide attempts and 96 (58.2%) cases of non-suicidal self-injury (NSSI). Males were predominant in both groups, comprising 46 (66.6%) of suicide attempters and 56 (58.3%) of NSSI cases. In terms of age distribution, suicide attempts were most frequent among individuals aged 31-40 years, accounting for 30 (43.5%), followed by the 18-30 and 41-50 years age groups, each with 16 (23.2%) cases. In contrast, NSSI was primarily observed in the 18-30 years age group, with 74 (77%), followed by 31-40 years with 12 (12.5%). Unmarried individuals were the most commonly affected, making up 32 (46.3%) of suicide attempters and 44 (45.8%) of NSSI cases. Regarding departmental referrals, the majority of suicide attempters, 29 (42%), were referred from the Medicine department, followed by Emergency Medicine. For NSSI cases, Emergency Medicine accounted for most referrals, with 73 (76%), while other departments contributed in smaller proportions. The most common method of self-harm among suicide attempters was the ingestion of harmful substances, seen in 33 (47.8%), followed by hanging in 25 (36.2%) cases. Among the methods of self-harm, ingestion of harmful substances was the most prevalent, affecting 71 individuals (73.9%), followed by the use of sharp objects in 22 cases (22.9%).

Table 2 shows that depressive disorder (n=80) was the most common diagnosis among all 165 patients with self-harm. Depressive disorders accounted for the highest number of suicide attempts, with 55 (68.7%) cases, while substance use disorder (n=76) was most frequent in NSSI cases, affecting 51 (67.1%) individuals. Personality disorders (n=29) were predominantly seen in NSSI, with 21 (72.4%) cases.

**Table 2:** Psychiatric diagnoses across patients with self-harm. Note: The total may exceed 165, as multiple diagnoses can occur in a single person. NSSI: Non-suicidal Self-Injury.

Psychiatric Diagnosis	With Suicidal Attempt (%)	With NSSI (%)	Total
Depressive Disorder	55 (68.7)	25 (31.3)	80
Substance Use Disorder	25 (32.9)	51 (67.1)	76
Personality Disorders	8 (27.6)	21 (72.4)	29
Psychotic Disorder	9 (75.0)	3 (25.0)	12
Others	2 (25.0)	6 (75.0)	8

Table 3 shows the association between suicide intent and a past history of suicide attempts, NSSI, and substance use.

**Table 3:** Relationship between intent of self-harm, past history of suicide and NSSI, and substance use. NSSI: Non-suicidal Self-Injury.

Variable	Suicidal Attempt (n=69)	NSSI (n=96)	Chi-square Value	P-value	OR
Past history of suicide	25 (36.2%)	12 (12.5%)	12.08	0.0006	3.98
Past history of NSSI	53 (76.8%)	79 (82.3%)	0.45	0.5024	0.71
With substance use	9 (13.0%)	51 (53.1%)	26.17	<0.001	0.13

A strong association was found between suicide intent and a past history of suicide attempts, with 36.2% of suicide attempters having a prior attempt compared to 12.5% of those with NSSI (p = 0.0006, OR = 3.98). In contrast, a history of NSSI was prevalent in both groups (76.8% in suicide attempters, 82.3% in NSSI cases); however, the difference was not significant (p = 0.5024), suggesting that while NSSI is often repetitive, it does not strongly predict future suicide attempts.

Substance use was significantly higher in NSSI cases (53.1%) than in suicide attempters (13.0%, p < 0.001, OR = 0.13), reinforcing its stronger link to impulsive self-harm rather than suicide attempts.

## Discussion

This study highlights key demographic and clinical differences between suicide attempters and individuals engaging in NSSI, providing insights into their distinct profiles and implications for mental health care. Table 1 indicates that NSSI (58.2%) is more prevalent than suicide attempts (41.8%). Males were predominant in both groups, more so among suicide attempters (66.6%) than in NSSI cases (58.3%), aligning with literature suggesting that men prefer lethal self-harm methods, whereas women are more likely to engage in non-lethal behaviors [6,7].

Age distribution reveals that suicide attempts are most prevalent among individuals aged 31-40, whereas NSSI is concentrated in the 18-30 age group. This supports evidence that NSSI often emerges in young adulthood due to emotional dysregulation and identity-related stressors [8,9]. Early-onset NSSI is associated with greater severity and frequency, emphasizing the need for early interventions. In contrast, suicide attempts in the 31-40 age group may be influenced by midlife stressors such as financial instability and relationship difficulties. A meta-analysis by Qin P et al. [10] highlights midlife suicide risks, particularly among adults aged 30-59, where socioeconomic factors, especially unemployment, play a significant role.

Marital status was another relevant factor, with unmarried individuals comprising the majority in both groups, 46.3% among suicide attempters and 45.8% in NSSI cases. The lack of social support in unmarried individuals is a well-documented risk factor for self-harm. Studies emphasize the protective nature of social support against suicide [11,12], underscoring the importance of assessing social networks when evaluating self-harm risk.

Referral patterns indicate that suicide attempters were predominantly referred from the Medicine department (42%), likely due to the severity of their attempts, such as poisoning or physical trauma. In contrast, NSSI cases were more frequently referred from Emergency Medicine (76%), reflecting the acute and repetitive nature of such behaviors. Given that the Emergency Medicine and Medicine departments handle most self-harm cases, targeted training for healthcare providers in these settings is essential. This highlights the importance of structured training of staff knowledge and the implementation of safety planning to reduce repeat suicide attempts [13]. It also underscores the importance of sensitizing other departments to screen for suicidal behavior, enabling early intervention.

Regarding self-harm methods, suicide attempters commonly ingested harmful substances such as pesticides and cleaning agents (47.8%), followed by hanging and burning, demonstrating a preference for lethal means. The high incidence of self-harm by ingestion can possibly be attributed to poor regulations on the sale of pesticides in India, making these toxic substances easily accessible. NSSI cases predominantly involved the ingestion of harmful substances (73.9%) or the use of sharp objects, consistent with patterns of repetitive, non-lethal self-harm. In contrast to the Western world, where firearms are a common method of self-harm, India's strict laws on arms and ammunition likely contribute to their absence as a means of self-injury. Restricting access to common means of self-harm, such as toxic substances and sharp objects, has been recommended as an effective prevention strategy [14,15,16].

Table 2 highlights the association between psychiatric diagnoses and self-harm behaviors. Depressive disorder was predominant among suicide attempters (68.7%), reinforcing its well-established role as a major suicide risk factor [17,18]. In contrast, only 31.3% of NSSI cases had depression, suggesting that while depressive symptoms may be present, NSSI often serves different psychological functions. Zhu X et al. [19] emphasize that NSSI increases the likelihood of developing suicidal ideation, reinforcing the need for early intervention in individuals with depressive symptoms.

Substance use disorder showed an inverse pattern, with NSSI cases (67.1%) outnumbering suicide attempters (32.9%). Substance use itself is associated with emotional dysregulation, which can have a bidirectional relationship with suicide behavior, both exacerbating distress and impairing impulse control. This supports research indicating that substance use often serves as a coping mechanism for emotional distress and is more common in self-injury than in suicide intent [20]. Integrating harm reduction strategies and psychoeducation into substance use treatments may help mitigate NSSI behaviors. Personality disorders were significantly more prevalent in NSSI cases (72.4%) than in suicide attempts (27.6%), aligning with research that identifies self-harm as a key characteristic of borderline and antisocial personality disorders [21,22]. For these individuals, self-harm often serves as an emotional regulation strategy rather than an indicator of suicide intent. Dialectical Behavior Therapy (DBT), which focuses on emotional regulation, has been particularly effective in reducing self-harm behaviors in this population.

Table 3 explores the relationship between past suicide attempts, NSSI, and self-harm intent. Suicide intent was significantly associated with a history of suicide attempts, with individuals having suicide intent nearly four times more likely to have previously attempted suicide (odds ratio = 3.98, p < 0.01). This aligns with research identifying prior suicide attempts as strong predictors of future suicide attempts [20,23]. Conversely, a history of NSSI was more prevalent among NSSI patients (79 out of 96) than among those with suicide intent (53 out of 69). Although the odds ratio of 0.71 suggests that individuals with suicide intent were slightly less likely to have an NSSI history, this difference was not statistically significant (p = 0.5024). While NSSI and suicide behaviors may overlap, their psychological motivations often differ [24,25]. Research indicates that approximately 70% of individuals who engage in NSSI attempt suicide at least once, with 55% having multiple attempts [26,27]. This highlights the importance of early intervention in NSSI cases to prevent escalation to suicide attempts and underscores the role of community-based mental health interventions in reducing self-harm behaviors by providing timely support. Preventive programs focusing on emotional regulation and stress management are particularly relevant given the high prevalence of NSSI.

Table 3 also shows that among individuals with suicide intent, only 13.04% reported substance use, with a significant association (Chi-Square = 26.17, p < 0.001). The odds ratio of 0.13 suggests that individuals with substance use were less likely to have suicide intent. In contrast, 53.13% of NSSI cases reported substance use, reinforcing the strong link between substance use and NSSI. Substance use frequently serves as a maladaptive coping mechanism for emotional distress, particularly in impulsive behaviors like NSSI. These findings contrast with studies such as Krishna M et al. (2014) [28], which reported varied substance use levels among self-harm patients. Sixty participants (86.9%) engaged in suicide behaviors without concurrent substance use, supporting findings by Grover S et al. (2016) [29], which highlight that many individuals engage in self-harm independently of substance use. While substance use is a significant risk factor, a substantial proportion of individuals engage in NSSI and suicide behaviors without it. Comprehensive screening for substance use disorders is essential in NSSI patients to integrate appropriate treatments, whereas interventions for suicide intent should prioritize addressing underlying psychiatric conditions over substance use.

This study was unique in Central India, but there were a few limitations. First, as a retrospective record-based analysis that lacked randomization, it relied on the accuracy and completeness of medical records, which could have led to missing or underreported data. Second, the study was limited to a single tertiary care hospital in Central India, which might not fully represent self-harm patterns in the broader population or rural settings. Third, it did not account for confounding factors such as family history of psychiatric illness, social support, or the role of impulsivity, all of which could have influenced self-harm behaviors. Additionally, the cross-sectional design prevented the establishment of causal inferences between self-harm behaviors, psychiatric disorders, and substance use. Future studies with prospective designs, larger sample sizes, and more comprehensive psychosocial assessments are needed to address these limitations and provide a deeper understanding of self-harm and its associated risk factors.

## Conclusions

This retrospective study underscores the critical link between NSSI, suicide attempts, and their shared risk factors. Identifying self-harm behaviors and treating them early could be the first step in managing potential suicide behaviors among those who engage in self-harm. The priority in the management of patients presenting at health facilities following suicide attempts is medical resuscitation and stabilization. As soon as the patient is medically stable, a thorough suicide risk assessment, which evaluates suicide ideation/intent, preceding circumstances, predisposing and protective factors, should be conducted. An assessment of current and ongoing suicide risk will assist in determining the safest place to manage the patient. Measures should be put in place to address the modifiable psychosocial risk factors for suicide, while appropriate pharmacotherapy is instituted for co-existing mental illness. Post-discharge care, such as referral to a psychologist, psychiatrist, or social worker, should be initiated by the primary care practitioner to ensure continuity of care. Lastly, we must not underestimate the gravity of self-harm and its association with suicide behaviors. By designing interventions tailored to each diagnostic group, mental health practitioners can better support patients and mitigate the burden of self-harm in clinical settings.

## Figures and Tables

**Figure 2:**
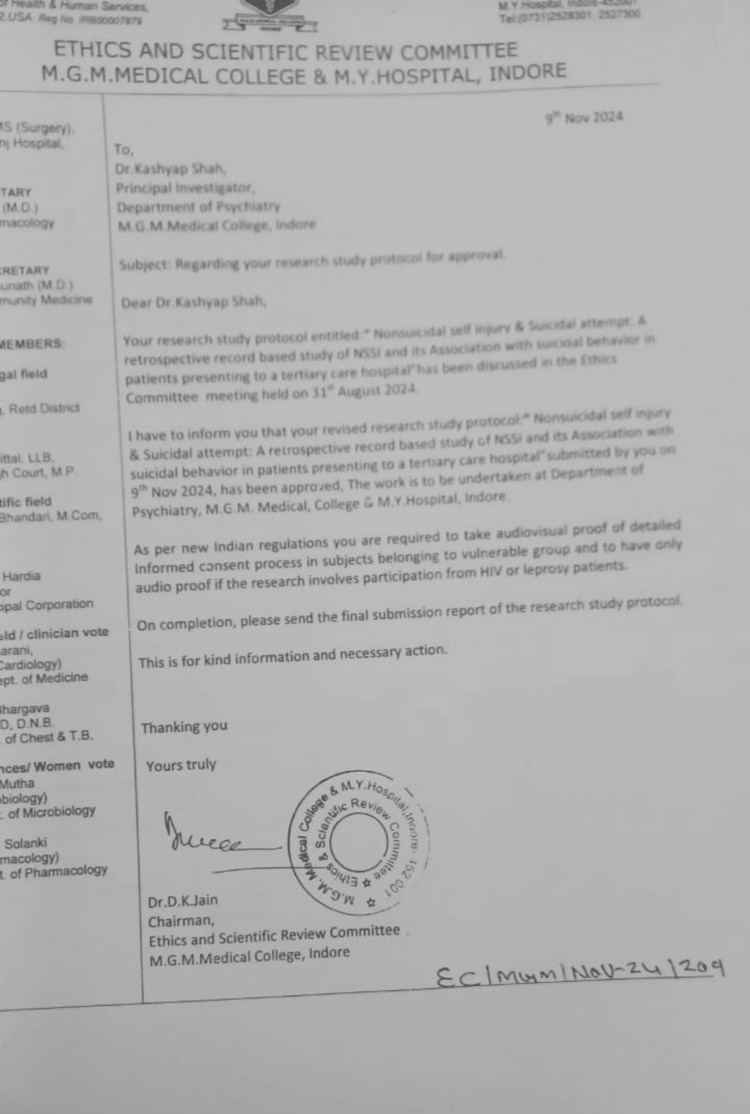
Ethics & Scientific Committee Approval Letter

## Appendices

Appendix 1
